# Intra-amniotic administration of l-glutamine promotes intestinal maturation and enteroendocrine stimulation in chick embryos

**DOI:** 10.1038/s41598-022-06440-z

**Published:** 2022-02-16

**Authors:** Naama Reicher, Tal Melkman-Zehavi, Jonathan Dayan, Zehava Uni

**Affiliations:** Department of Animal Science, The Robert H. Smith, Faculty of Agriculture, Food and Environment, The Hebrew University of Jerusalem, 76100 Rehovot, Israel

**Keywords:** Gastrointestinal models, Cell growth, Differentiation

## Abstract

Initial nutritional stimulation is a key driving force for small intestinal maturation. In chick embryos, administration of l-glutamine (Gln) into the amniotic fluid stimulates early development of the small intestinal epithelium by promoting enterocyte differentiation. In this study, we evaluated the effects of intra-amniotic administration of Gln on enterocyte morphology and function, and elucidated a potential enteroendocrine pathway through which Gln stimulates small intestinal maturation. Our results show that Gln stimulation at embryonic day 17 significantly increased enterocyte and microvilli dimensions by 10 and 20%, respectively, within 48 h. Post-hatch, enterocytes and microvilli were 20% longer in Gln-treated chicks. Correspondingly, Gln stimulation significantly upregulated mRNA expression of brush border nutrient transporters PepT-1 and SGLT-1 and tight junction proteins TJP-1 and TJP-2, before and after hatch (*P* < 0.05). Since GLP-2 signaling from intestinal L-cells is associated with enterocyte growth, functionality and integrity, we examined the effects of Gln stimulation on mRNA expression of key hormones and receptors within this enteroendocrine pathway and found significant increases in GLP-2R, IGF-1 and IGF-1R expression before and after hatch (*P* < 0.05). In conclusion, our findings link primary nutrient stimulation in the developing small intestine with enterocyte morphological and functional maturation and enteroendocrine signaling.

## Introduction

Small intestinal maturation in chicken occurs several days before and after hatch, during the nutritional transition from egg yolk nutrients to exogenous feeding^1–3^. Throughout this period, the small intestinal mucosa gains functionality through cellular growth, proliferation and differentiation of enterocytes^4,5^, as well as expansion of enterocyte brush border surface areas through microvilli assembly^6,7^.

Initial post-hatch feeding is a key stimulatory factor for these processes^8–11^, yet nutritional stimulation of the small intestine begins *in-ovo*, from embryonic day 17 (E17), when the embryo starts ingesting its amniotic fluid^1,2,12,13^. A method developed for enriching the amniotic fluid with nutrients (in-ovo feeding), has been widely used for examining the effects of pre-hatch nutritional stimulation on development of the small intestine^13^. Various studies have shown that intra-amniotic administration of specific nutrients, such as proteins, amino acids and carbohydrates, upregulates the expression and activity of nutrient transporters and digestive enzymes, and expands the digestive and absorptive surface area of the small intestine before and after hatch^14–18^.

Recently, intra-amniotic administration of l-glutamine (Gln) was found to promote proliferation and differentiation in the small intestinal epithelium of chick embryos, resulting in higher quantities of enterocytes during the first week post-hatch^19^. The objectives of this current study were to evaluate the effects of intra-amniotic administration of Gln on enterocyte morphology and function and to examine a potential mechanism by which Gln affects these parameters.

Previous studies in mammals showed that luminal nutrients, and Gln in particular, bind to enteroendocrine L-cells, which in turn initiate paracrine hormonal signaling^20–23^. Intestinal hormones, such as CCK, GIP, PYY and GLP-1, control intestinal functionality, metabolism and feed intake^24^. However, induction of intestinal epithelial proliferation and differentiation is specifically associated with GLP-2 signaling^21^. GLP-2 is secreted by nutrient-stimulated enteroendocrine cells into the lamina propria, where it binds to its receptor (GLP-2R), located on intestinal sub-epithelial myofibroblasts (ISEMFs)^21,25,26^. ISEMFs then secrete Insulin-like Growth Factor 1 (IGF-1), which binds to its receptor, IGF-1R, on enterocyte basolateral membranes, and initiates subsequent effects^20,27^. These include increases in enterocyte dimensions and elongation of microvilli^28^; improved enterocyte functionality through upregulation of di-tri Peptide transporter 1 (PepT-1) and Sodium Glucose Transporter1 (SGLT-1) expression (demonstrated in both mammals and chicken)^29,30^ and enhanced expression of tight junction proteins, such as TJP-1, TJP-2 and Occludin, which are required for mucosal barrier function^31,32^.

We therefore hypothesized that intra-amniotic administration of Gln will induce the growth of enterocytes and their apical brush border and enhance nutrient transporter and tight junction gene expression through L-cell stimulation.

To test this hypothesis, we conducted intra-amniotic administration of Gln (1% in 0.4% NaCl), and its diluent, NaCl (adjusted to 0.75% for equal osmolarity), in E17 chick embryos, as described previously^11,13,17,19^. We examined the effects of these treatments, compared to a non-administered control group, on pre- and post-hatch enterocyte maturation by measuring jejunal enterocyte and microvilli dimensions and quantifying mRNA expression of nutrient transporters PepT-1, SGLT-1 and tight junction proteins TJP-1, TJP-2 and Occludin. We then evaluated the involvement of L-cell signaling in Gln-induced enterocyte maturation, by visualizing intestinal GLP-2 immunoreactive cells and examining the expression of genes encoding PGB (Proclucagon, the GLP-2 precursor), GLP-2R, IGF-1 and IGF-1.

## Results and discussion

**Figure 1:**
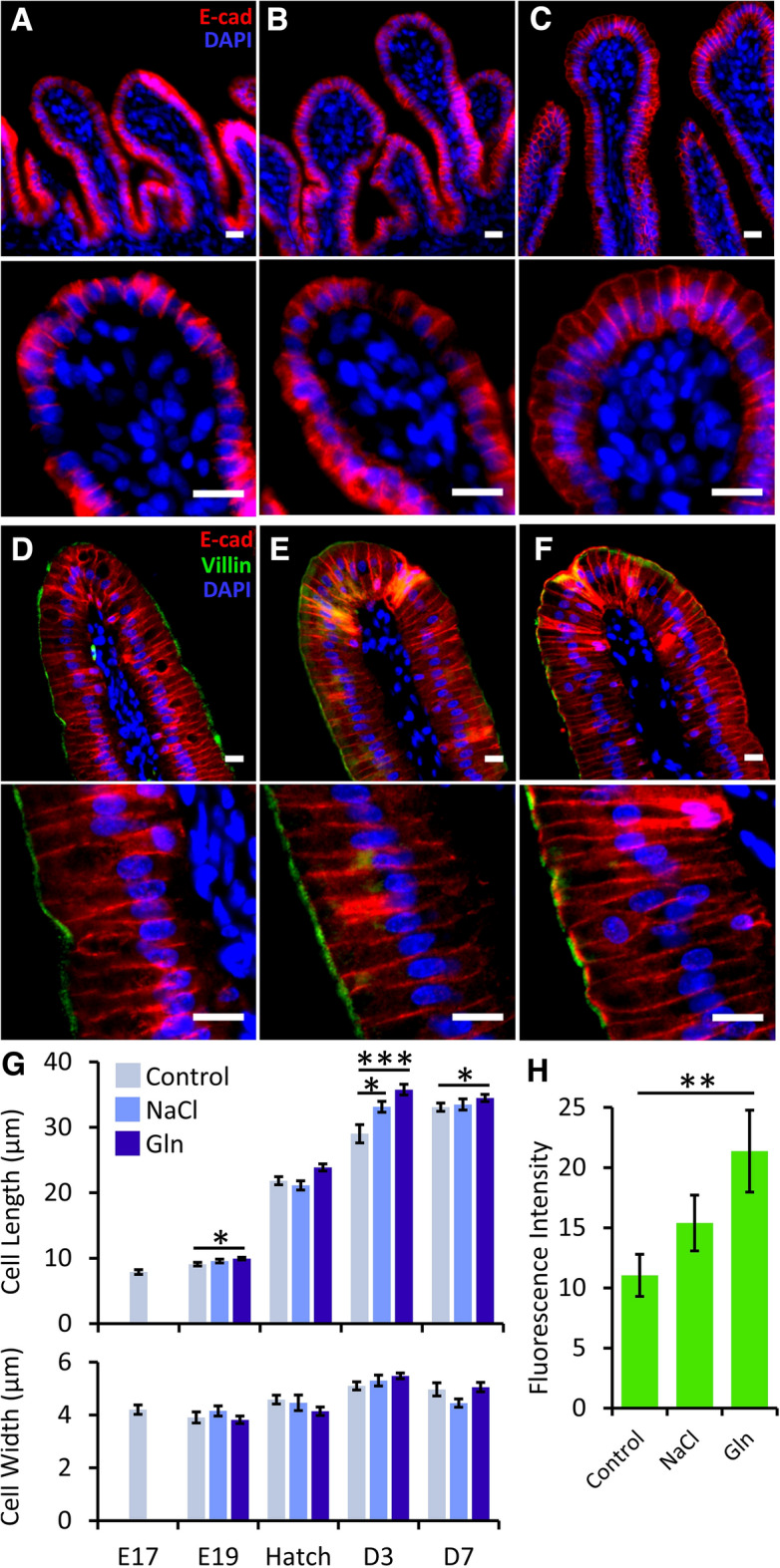
Intra-amniotic administration of l-glutamine enhances enterocyte growth before and after hatch. (**A**–**C**) Representative images of E-cadherin (E-cad) (red) and nuclear DAPI immunostaining at embryonic day 19 (E19) in Control (**A**), NaCl—(**B**) and Gln—(**C**) treated embryos. Images were captured at X20 magnification. Bottom panels are X40 magnifications of villi tips. (**D**–**F**) Representative images of E-cad (red), Villin (green) and nuclear DAPI (blue) immunostaining at post-hatch day 3 (D3) in Control (**D**), NaCl—(**E**) and Gln—(**F**) treated chicks. Images were captured at X20 magnification. Bottom panels are X40 magnifications of enterocytes. Scale bars, 10 µm. (**G**) Enterocyte lateral lengths and apical widths before and after hatch in Control, NaCl- and Gln-treated embryos/chicks. (**H**) Fluorescence intensity of Villin immunostaining at D3 in Control, NaCl- and Gln-treated chicks. Image analysis was conducted in FIJI software (https://imagej.net/software/fiji/). Data are means ± standard error means from measurements of 10 cells from 8 villi tips in 3 individual embryos/chicks per group, at each timepoint. Asterisks mark significant differences from Control embryos/chicks at each timepoint by t-test: *P < 0.05, **P < 0.001, ***P < 0.001.

The effects of intra-amniotic administration of 1% l-glutamine (Gln) and 0.75% NaCl (NaCl) on jejunal enterocyte morphometric maturation before and after hatch were examined by visualizing cellular outlines through immunostaining of the adherens junction molecule E-Cadherin (E-cad), combined with the microvilli marker Villin and DAPI nuclear staining (Fig. 1). Enterocyte lengths increased 4.3-fold between E17 and D7 in all groups, and significant differences were observed between groups at E19, hatch and D3 (*P* = 0.03, *P* = 0.01, *P* = 0.0003, respectively) (Fig. 1G). Compared to Control embryos, enterocytes of Gln-treated embryos were 10% longer at E19 and hatch (*P* = 0.01), while enterocytes of NaCl-treated embryos did not differ significantly (Fig. 1A–C,G). At D3, enterocytes of both Gln and NaCl-treated chicks were significantly longer than those of Control embryos (by 14%, *P* = 0.01, and by 20%, *P* = 0.0002, respectively) (Fig. 1D–G). By D7, enterocytes of Control chicks reached similar lengths to those of Gln and NaCl-treated chicks, which did not portray further increases in comparison to D3 (Fig. 1G). Enterocyte widths remained stable within groups at all pre- and post-hatch ages (Fig. 1G, bottom graph). Therefore, the observed increases in their lengths in the Gln group generated a more columnar epithelium at an earlier age. Since the intestinal epithelium of chicks reaches morphological maturity during the first week post-hatch^4^, we conclude that intra-amniotic administration of Gln promoted early onset of enterocyte growth, achieving maximal cellular columnarity at D3, rather than D7.

**Figure 2:**
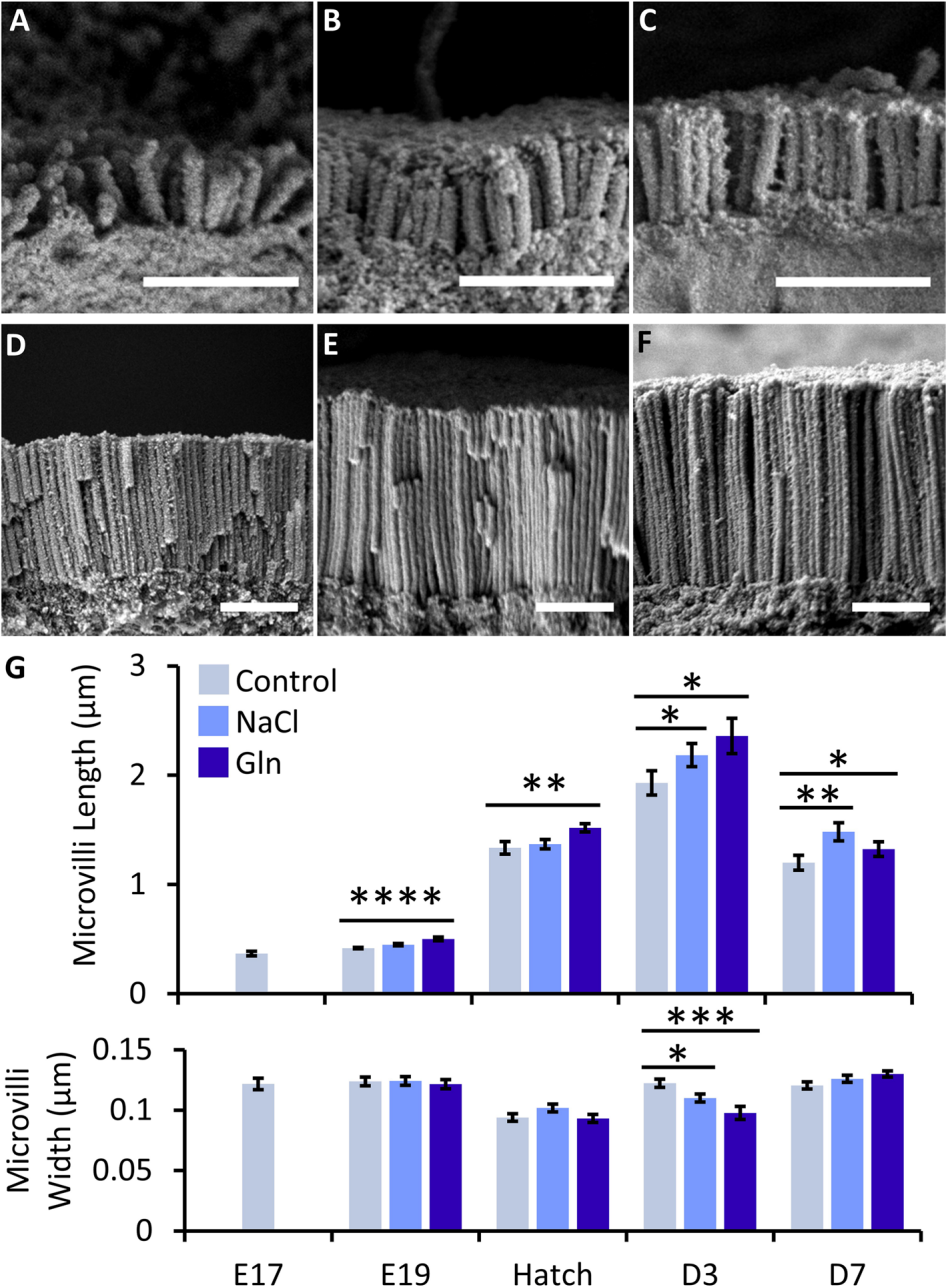
Intra-amniotic administration of l-glutamine enhances microvilli growth before and after hatch. (**A**–**C**) Representative scanning electron microscope (SEM) images of microvilli lengths at embryonic day 19 (E19) in Control (**A**), NaCl—(**B**) and Gln—(**C**) treated embryos. (**D**–**F**) Representative SEM images of microvilli lengths at post-hatch day 3 (D3) in Control (**D**), NaCl—(**E**) and Gln—(**F**) treated chicks. Scale bars, 1 µm. (**G**) Microvilli lengths and widths before and after hatch in Control, NaCl and Gln-treated embryos/chicks. Image analysis was conducted in FIJI software. Data are means ± standard error means from measurements of 10 microvilli from 5 villi tips in 3 individual embryos/chicks per group, at each timepoint. Asterisks mark significant differences compared to Control embryos/chicks at each timepoint by t-test: *P < 0.05, **P < 0.01, ***P < 0.001, ****P < 0.0001.

Villin immunostaining was confined was to enterocyte brush borders, and was barely detectable at pre-hatch ages. Post-hatch, Villin immunostaining was most prominent at D3 (representative images in Fig. 1D–F, bottom panel), and portrayed significant differences in signal intensities between groups (*P* = 0.03), with a 1.9-fold increase in the Gln group, compared to the Control group (*P* = 0.008) (Fig. 1H). In light of this result, we sought to visualize microvilli ultrastructure in high-resolution scanning electron microscopy (SEM) for examining the effects of Gln and NaCl stimulation on brush border development (Fig. 2). Results showed that at E19, microvilli of both NaCl and Gln-treated embryos were more perpendicularly oriented to enterocyte apical surfaces, compared to microvilli of Control embryos (Fig. 2A–C). This indicates advanced brush border development, since pre-hatch microvilli straighten with age through rootlet elongation and terminal web connection^33^.

Microvilli lengths significantly differed between groups at E19 (*P* < 0.0001), hatch (*P* = 0.013), D3 (*P* = 0.019) and D7 (*P* < 0.0001) and microvilli widths significantly differed between treatment groups at D3 only (*P* = 0.0003). At E19, a 20% increase in microvilli lengths was measured Gln-treated embryos, compared to Control embryos (*P* < 0.0001), while microvilli lengths in NaCl-treated embryos did not differ from Control embryos (Fig. 2G). Similarly, microvilli at hatch were 14% longer in Gln-treated chicks, compared to Control chicks (*P* = 0.009), and no differences were found between NaCl-treated and Control chicks (Fig. 2G). At D3, brush borders in both treatment groups portrayed advanced development in comparison to Control chicks, as microvilli of NaCl-treated chicks were 13% longer (*P* = 0.045), and microvilli of Gln-treated chicks were 22% longer (*P* = 0.018), reaching peak values of 2.2 and 2.4 µm, respectively. Furthermore, microvilli widths at D3 were significantly reduced in NaCl and Gln-treated chicks (by 10%, *P* = 0.029 and 20%, *P* = 0.0003, respectively) compared to Control chicks (Fig. 2D–G). Since microvilli organization was similar in all groups at this timepoint (Fig. 2D–F), a reduction in their widths enables higher microvilli density.

Based on our previous findings, microvilli reach maximal lengths at D3 and significantly amplify enterocyte apical surface areas through their dimensions and densities^6^. Our current results indicate that intra-amniotic administration of Gln enhanced pre-hatch brush border developmental patterns, thus dramatically expanding enterocyte surface areas at D3.

At D7, microvilli followed the same pattern reported in our previous work^6^, as their lengths decreased by 38, 32 and 44% in Control, NaCl and Gln treated chicks, respectively. However, microvilli of NaCl and Gln-treated chicks remained significantly longer (by 23%, *P* = 0.008 and 10%, *P* = 0.034, respectively) than microvilli of Control chicks at D7 (Fig. 2G). This finding indicates possible long-term increases in brush border surface areas following Gln stimulation, thus significantly contributing to enterocyte absorptive capacities.

**Figure 3:**
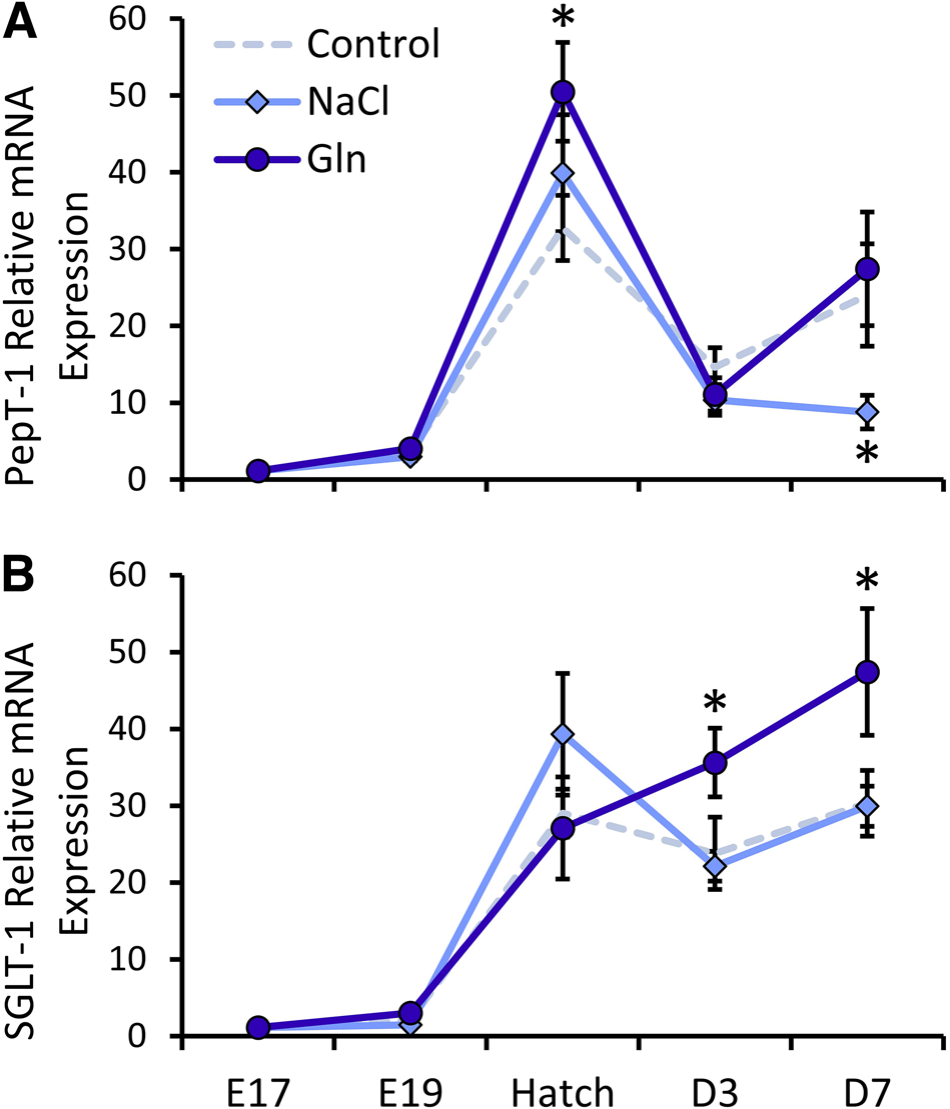
Intra-amniotic administration of l-glutamine increases brush border nutrient transporter gene expression after hatch. Real-Time qPCR analysis of (**A**) Peptide transporter 1 (PepT1) and (**B**) Sodium Glucose Transporter 1 (SGLT-1) at embryonic day 19 (E19), hatch, post-hatch day 3 (D3) and D7. Expression in each treatment group and each timepoint is normalized to β-actin expression and relative to mean expression at E17 by the 2^−ΔΔCt^ method (further explained in “Methods”). Data are means ± standard error means from jejunal samples of 6 embryos/chicks per group at each timepoint. Asterisks indicate significant differences from Control embryos/chicks at each timepoint by t-test: *P < 0.05.

Since our results revealed distinct morphological maturation of microvilli, we evaluated the effects of intra-amniotic administration of Gln and NaCl on brush border nutrient transporter mRNA expression before and after hatch, as an indicative factor of enterocyte functionality. This was conducted by a Real-Time qPCR analysis of Peptide transporter 1 (PepT-1) and Sodium Glucose Cotransporter 1 (SGLT-1) (Fig. 3).

In the Control group, expression of both genes exhibited a dramatic 30-fold increases between pre- and post-hatch ages, in accordance with previous findings^34–36^ (Fig. 3A,B). Significant differences between groups were found for PepT-1 expression at hatch (*P* = 0.027) and D7 (*P* = 0.02), and for SGLT-1 expression at D3 and D7 (*P* = 0.048). The surge in PepT-1 expression at hatch was 54% higher in Gln-treated chicks, compared to Control chicks (*P* = 0.021), while expression in NaCl-treated chicks did not differ significantly from Control chicks. At D3 and D7, PepT-1 expression decreased in all groups, in accordance with previous reports^36^ and did not differ between Gln-treated chicks and Control chicks at these ages. However, NaCl-treated chicks exhibited a 63% decrease in PepT-1 expression at D7, compared to Control chicks (*P* = 0.036) (Fig. 3A).

In contrast, SGLT-1 expression was similar in all groups at hatch. However, Gln-treated chicks exhibited significant, 50 and 57% increases in SGLT-1 expression in comparison to Control chicks at D3 and D7, respectively (*P* = 0.042), while expression in the NaCl group did not differ from the Control group at these timepoints. We conclude that intra-amniotic administration on Gln, but not NaCl, significantly improved the absorptive potential of di- and tri-peptides and glucose after hatch, corresponding to the enhancements in brush border morphometric parameters.

**Figure 4:**
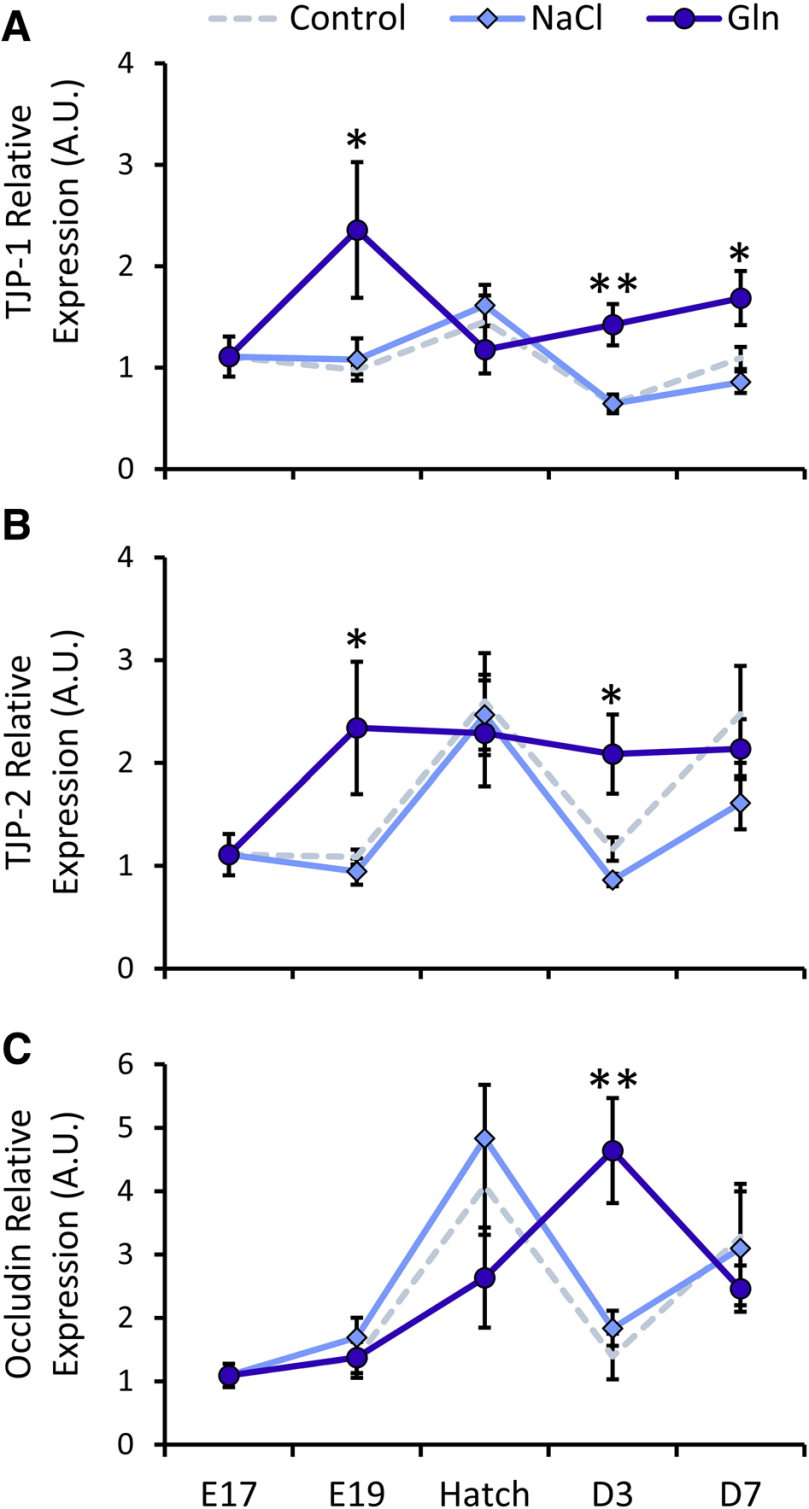
Intra-amniotic administration of l-glutamine enhances tight junction gene expression after hatch. Real-Time qPCR analysis of (**A**) tight junction protein 1 (TJP-1) (**B**) TJP-2 and (**C**) Occludin at embryonic day 19 (E19), Hatch, post-hatch day 3 (D3) and D7. Expression in each treatment group is normalized to β-actin expression and relative to expression in the Control group at E17 by the 2^−ΔΔCt^ method (further explained in “Methods”). Data are from jejunal samples of 6 embryos/chicks per group at each timepoint. Asterisks indicate significant differences from Control embryos/chicks at each timepoint by t-test: *P < 0.05, **P < 0.01.

An additional crucial factor for intestinal maturation is the epithelial barrier, generated by tight junctions, which develop and mature before and after hatch^37^. Additionally, tight junction formation is a key regulatory factor in brush border development^33,38,39^. We therefore examined the effects of intra-amniotic administration of Gln and NaCl on the expression of tight junction proteins 1 and 2 (TJP-1 and TJP-2, respectively), and the associated transmembrane protein Occludin^40^ (Fig. 4).

Results showed that at E19, there were significant differences between groups in both TJP-1 and TJP-2 expression (*P* = 0.023, *P* = 0.013, respectively). Gln-treated embryos exhibited significant, 2.1-fold increases in expression of both genes, compared to Control embryos (*P* = 0.032, *P* = 0.041, respectively) (Fig. 4A,B). Post-hatch, TJP-1 expression differed between groups at D3 (*P* = 0.001) and D7 (*P* = 0.016). Expression was 2.2-fold higher at D3 (*P* = 0.003) and 1.5-fold higher at D7 (*P* = 0.045) in Gln-treated chicks, compared to Control chicks, while expression did not differ between NaCl-treated chicks and Control chicks (Fig. 4A). Post-hatch TJP-2 expression differed between groups at D3 (*P* = 0.0075). Expression was 1.79-fold higher (*P* = 0.031) in Gln-treated chicks and 27% lower in NaCl-treated chicks, compared to Control chicks (Fig. 4B). Occludin expression also differed between groups at D3 (*P* = 0.0014), displaying a 3.3-fold increase in expression in Gln-treated chicks, compared to Control chicks (*P* = 0.002), while expression did not differ between NaCl-treated and Control chicks (Fig. 4C).

Taken together, intra-amniotic administration of Gln enhanced intestinal developmental dynamics by promoting enterocyte growth, nutrient transporter expression and tight junction formation, thus improving post-hatch nutrient absorption and intestinal integrity. These effects were restricted to the first week post-hatch, after which the small intestinal epithelium reaches maturity^4,10^.

In light of these results, we sought to determine the mechanism by which the administered Gln stimulated the developing small intestinal cells to initiate the observed effects. Various studies in mammals linked enhancements in intestinal morphology, function and integrity to GLP-2 secretion from enteroendocrine L-cells^28–32^, and Gln is a known stimulatory factor for this pathway^21–23^. We therefore hypothesized that the observed effects on enterocyte maturation were a result of Gln-mediated L-cell stimulation. To test this hypothesis, we first identified and characterized jejunal L-cells at pre- and post-hatch ages.

L-cells in mammals are most abundant in the ileum, and are distinguishable from enterocytes by their unique morphology and the presence of various secretory granules within their cytoplasm^41^. In chicken, L-cells were identified in the ileum at D7 by Glucagon-like Peptide 2 (GLP-2) immunoreactivity through fluorescence and transmission electron microscopy (TEM)^42^, and were also found with decreased abundancy along jejunum^43^. We therefore visualized L-cells within our jejunal samples at pre- and post-hatch ages by GLP-2 immunostaining.

**Figure 5:**
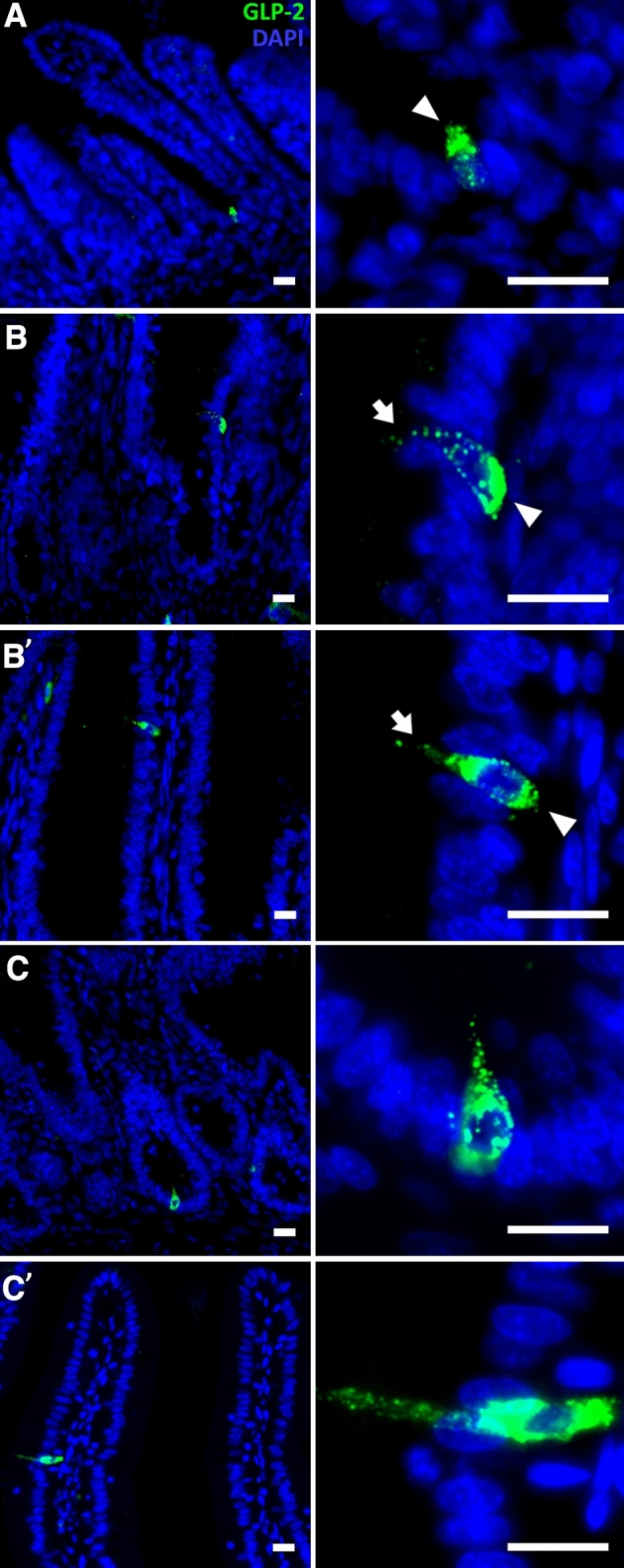
Morphology and localizations of jejunal L-cells before and after hatch. Representative images of glucagon-like peptide 2 (GLP-2) (green) and nuclear DAPI (blue) immunostaining in Control embryos and chicks at embryonic day 19 (E19) (**A**), hatch (**B**, **B′**) and post-hatch day 7 (D7) (**C**, **C′**). At E19, GLP-2 immunoreactive cells were located at villi bottoms (**A**) with higher signal intensity at the apical pole (right panel, arrowhead). At hatch, GLP-2 immunoreactive cells were located at upper crypt regions (**B**) and within the villus epithelium (**B′**), and displayed higher signal intensities at the basolateral pole (right panels, arrowheads) and lower apical signal intensity, outlining a thin cytoplasmatic process (right panels, arrows). At D7, GLP-2 immunoreactive cells had similar morphology, and were found throughout the crypt and villus epithelium, including crypt bottoms (**C**) and villi tips (**C′**). Left panel images were magnified X20 and right panel images were magnified X40. Scale bars, 10 µm. Image analysis was conducted in FIJI software.

Though no L-cells were observed at E17, rare occurrences of L-cells were noted at E19 (≤ 1 per complete cross section, n = 6) (Fig. 5A). L-cells at E19 were restricted to villi bottoms and GLP-2 signal intensity was higher within the apical pole of the cell (Fig. 5A, right panel, arrowhead), outlining an oval cell structure. This is in contrast to adult ileal L-cells in chicken, which portray increased GLP-2 signal intensity within the basolateral pole, and possess a thin cytoplasmatic process extending into the intestinal lumen^42,43^. At hatch, L-cells were found at upper crypt regions (Fig. 5B) as well as along the villus epithelium (Fig. 5B′). Their morphology resembled that of L-cells at D7 in the jejunum (Fig. 5C,C′) and ileum^42,43^ as well as adult mice ileal L-cells^41^.

These results reveal that the jejunal L-cell population appears between E17 and E19, when the embryo ingests its amniotic fluid^1,2,12,13^, and fully matures after hatch, when exogenous feeding begins. This indicates synchronization between the development of enteroendocrine function in the small intestine and primary nutritional stimulation in chick embryos, as has been described in mammals^44^.

**Figure 6:**
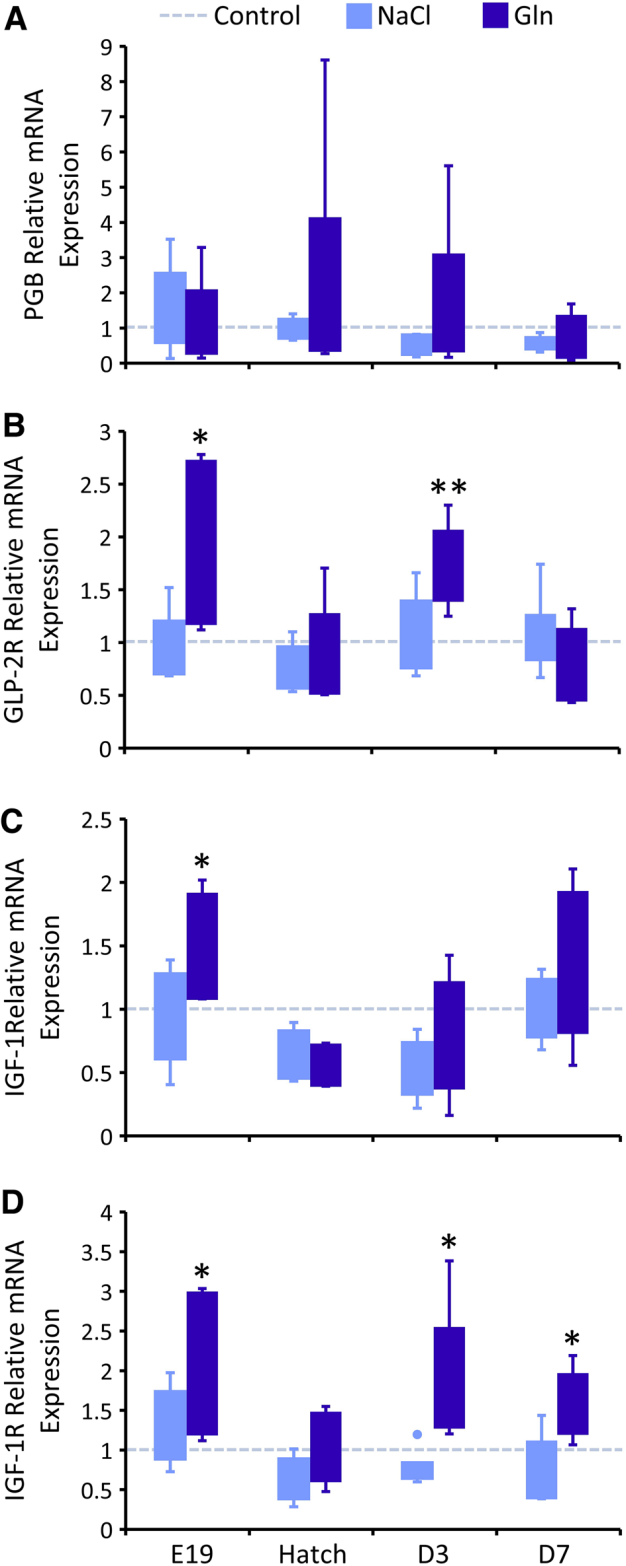
Intra-amniotic administration of l-glutamine enhances GLP-2 signaling gene expression after hatch. Real-Time qPCR analysis of (**A**) glucagon like protein 2 (GLP-2) precursor Proglucagon B; (**B**) GLP-2 receptor; (**C**) Insulin-like growth factor-1 (IGF-1); (**D**) IGF-1 receptor at embryonic day 19 (E19), hatch, post-hatch day 3 (D3) and D7. Expression in each treatment group is normalized to β-actin expression and relative to expression in the Control group at each timepoint (set as 1, dotted line) by the 2^−ΔΔCt^ method (further explained in “Methods”). Data are from jejunal samples of 6 embryos/chicks per group at each timepoint. Asterisks indicate significant differences from Control embryos/chicks at each timepoint by t-test: *P < 0.05, **P < 0.01.

In order to examine whether intra-amniotic administration of Gln and NaCl affected jejunal L-cell development, GLP-2 reactive cell abundances and staining intensities were measured in all groups at E19, hatch, D3 and D7. There were no significant differences in these parameters between groups at all examined timepoints (most likely due to their low abundancies, 3–5 per complete cross section, n = 6). Therefore, we proceeded to investigate the effects of Gln and NaCl administration on L-cell stimulation by examining the expression of genes encoding key components of L-cell signaling. Genes examined were Proglucagon B (PCB), which gives rise to GLP-2 through tissue-specific alternative splicing in chicken^45,46^, GLP-2 Receptor (GLP-2R), Insulin-like Growth Factor 1 (IGF-1) and IGF-1 Receptor (IGF-1R). Results revealed a tendency of increased expression of PCB at hatch and D3 in Gln-treated chicks, but this effect was not statistically significant due to high variability between individual Gln-treated chicks (Fig. 6A). Similarly, previous studies in chicken reported that fasting and refeeding did not affect intestinal PCB expression^45^. It may therefore be possible that the effects of intra-amniotic administration of Gln on GLP-2 synthesis occur at the post-translational level, or downstream.

However, expression of GLP-2R differed between groups at E19 (*P* = 0.042) and D3 (*P* = 0.0159). Expression in the Gln group was higher by 75% (*P* = 0.072) and 62% (*P* = 0.006) than in the Control group at E19 and D3, respectively (Fig. 6B). Concurrently, IGF-1 expression at E19 also differed between groups (*P* = 0.028), due to a 47% increase in expression in Gln-treated embryos, compared to Control embryos (*P* = 0.041) (Fig. 6C). These results may indicate activation of ISEMFs to synthesize and secrete IGF-1 in response to GLP-2 binding. The effects of IGF-1 on enterocytes are mediated by its receptor, IGF-1R^27,28^, and its expression differed significantly between groups at E19, D3 and D7 (*P* = 0.022, *P* = 0.0148, *P* = 0.0415, respectively). This was due to significant increase in expression in Gln-treated, compared to Control embryos and chicks (by 86% at E19, *P* = 0.029), (by 88% at D3, *P* = 0.031) and (by 58% at D7, *P* = 0.01) (Fig. 6D). Expression of these genes in NaCl-treated embryos and chicks did not differ from the Control group at all ages (Fig. 6A–D).

Taken together, these results demonstrate pre- and post-hatch upregulation of key components in L-cell paracrine hormonal signaling, in response to pre-hatch luminal Gln, thus linking the effects of Gln stimulation on enterocyte maturation with L-cell stimulation.

Though intra-amniotic administration of NaCl elicited some beneficial effects on enterocyte maturation, these effects were limited to post-hatch morphometric parameters. Since amniotic enrichment with NaCl was previously shown to promote proliferation in the small intestinal epithelium through activation of ion-dependent nutrient transporters during amniotic fluid ingestion^19^, it may be possible that NaCl promoted earlier morphological maturation of enterocytes, but did not stimulate L-cell signaling, and thus failed to enhance absorptive and barrier functions.

**Figure 7:**
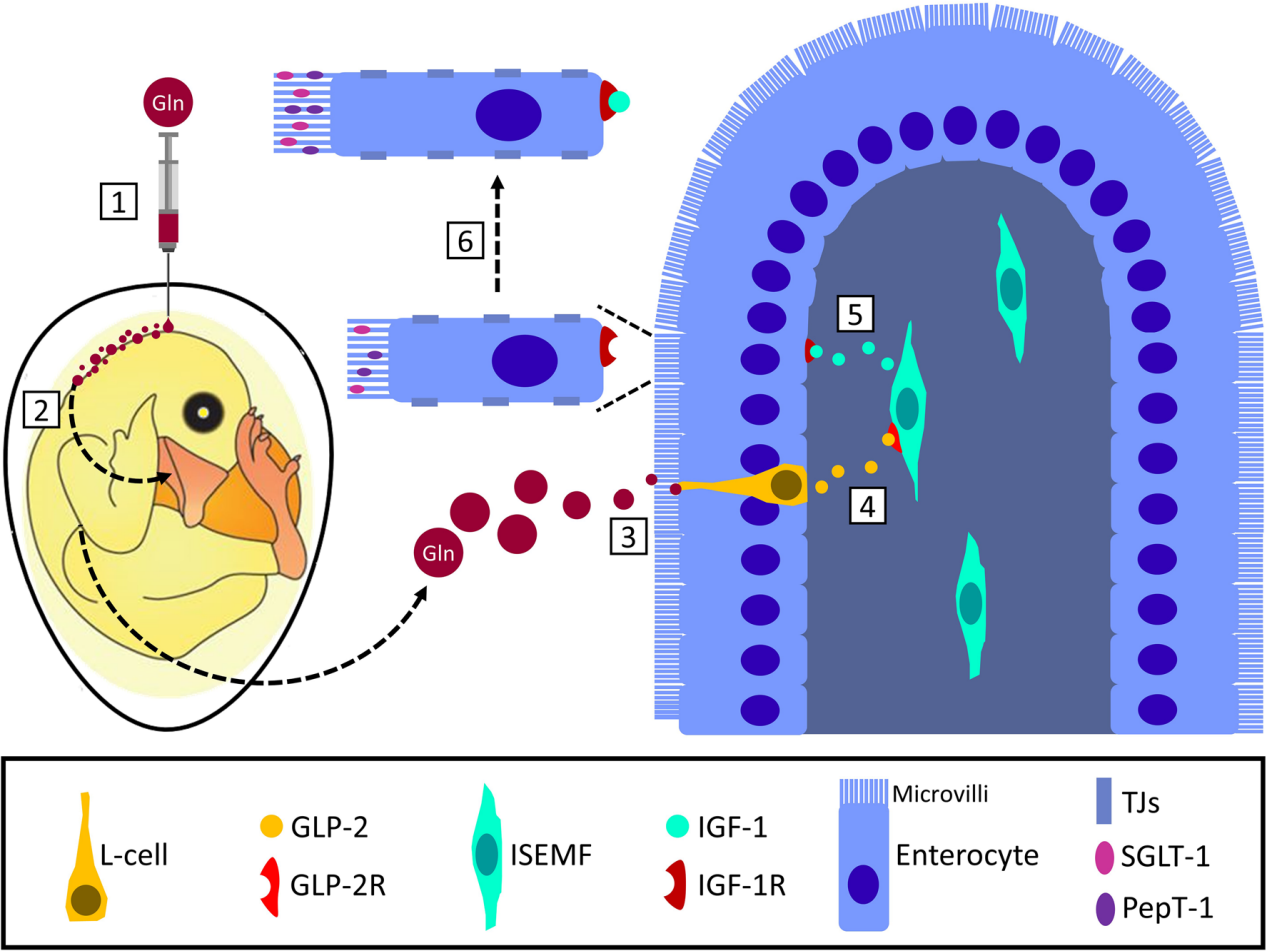
Proposed mechanism of action for l-glutamine-mediated enterocyte maturation through L-cell signaling. (1) Gln is administered into the amniotic fluid of the developing chick embryo; (2) the chick embryo ingests the enriched amniotic fluid before hatch; (3) in the small intestine, the administered Gln binds to epithelial L-cells; (4) L-cells secrete GLP-2 into the lamina propria in response to Gln stimulation, and GLP-2 binds to GLP-2R on ISEMFs; (5) ISEMFs secrete IGF-1 in response to GLP-2 binding, and IGF-1 binds to IGF-1R on enterocyte basolateral membranes; (6) IGF-1 stimulates increases in enterocyte dimensions, microvilli lengths, nutrient transporter expression and tight junction expression. This figure was prepared using Adobe Illustrator (version 21.0) and Microsoft PowerPoint Professional Plus 2016. Abbreviations: Gln, L-glutamine; GLP-2, Glucagon-like Peptide 2; GLP-2R, Glucagon-like Peptide 2 Receptor; ISEMF, intestinal sub-epithelial myofibroblast; IGF-1, Insulin-like Growth Factor 1; IGF-1R, Insulin-like Growth Factor 1 Receptor, TJs, tight junctions; SGLT-1, Sodium-Glucose Cotransporter 1; PepT-1, Peptide Transporter 1.

In conclusion, intra-amniotic administration of Gln enhanced enterocyte morphology, function and integrity during the critical first week post-hatch. These effects may be attributed to several pathways, including increased cellular energy due to Gln uptake and secretion of various intestinal hormones associated with enhanced intestinal functionality. Our results show that Gln-induced paracrine GLP-2 signaling, which has been widely associated with the effects described in this study^20–22,27,28^, as well as increased proliferation and differentiation found in our previous study^19^. Although direct evidence for this pathway has not been validated, we are the first to present a link between pre-hatch nutrient sensing and post-hatch enhancements in the developmental dynamics of small intestine of chicks. Figure 7 depicts the major findings in this study and a proposed mechanism of action. To date, studies of intestinal chemosensation in chicken have been limited to characterization and localization of enteroendocrine cells and their taste receptors^42,43,47–49^. Further investigation of chicken L-cell characteristics and function is needed in order to shed light on the connection between intestinal nutrient sensing on absorption and satiety, as well as the gut-brain connection in chicken, which has been established in mammals^50,51^. Nevertheless, our findings demonstrate that activation of pre-hatch intestinal chemosensation and subsequent hormonal signaling is possible through intra-amniotic administration of specific nutrients, and results in improved intestinal morphology and function. We therefore provide a model for investigating the link between primary nutrient sensing and intestinal maturation, as well as an applicative method for shaping intestinal development in chicken.

## Methods

### Ethics declarations

All animal experiments were conducted following guidelines for the care and use of laboratory animals of the Israel Ministry of Health and in accordance with ARRIVE guidelines. All methods performed were approved by the Hebrew University Institutional Animal Care and Use Committee (IACUC), approval number: AG-17-15355-2.

### Incubation and intra-amniotic nutrient administration

All animal experiments were conducted in accordance with ARRIVE guidelines and approved by the Hebrew University Institutional Animal Care and Use Committee (IACUC, license number: AG-17-15355-2). Fertile broiler eggs (Cobb 500, n = 150) were obtained from a commercial hatchery (Brown Ltd., Hod-Hasharon, Israel) and incubated in a Petersime hatcher at the Faculty of Agriculture of the Hebrew University, under standard conditions (37.8 °C, 60% relative humidity). Egg viability was examined by candling at the 14th day of incubation (E14) and unviable eggs were discarded. At E17, eggs were divided into three treatment groups of equal weights (n = 45 each) and placed at room temperature (RT). Intra-amniotic administration of was conducted as described previously^11^. Briefly, all eggs were disinfected by spraying of 75% ethanol. In two groups, a hole was punched at the wide egg pole, through which a 21-gauge, 1.25 inch needle was vertically inserted and a 0.6 ml sterile nutrient solution was injected into the amniotic fluid. Solutions consisted of 1% (wt/vol) l-glutamine (Sigma Aldrich, Rehovot, Israel) in 0.4% NaCl in the Gln group, and 0.75% NaCl in the NaCl group. Eggs of the Control group were not injected. Injected and Control eggs were sprayed again with 75% ethanol and placed in hatching trays inside the hatcher. Hatching occurred between E20 and E21 with no significant differences in hatching time and percentage between groups (mean hatchability was 94.3%). Chicks that hatched between E20.5 and E21 from all groups were marked according to their treatment group and housed together in a brooder with ad-libitum access to water and commercial starter feed (22% crude protein, 5.8% ash, 3035 kcal kg^−1^; Y. Brown & Sons Feedmill, Hod HaSharon, Israel).

### Tissue sampling

For embryonic tissue sampling, 6 embryos were randomly selected prior to intra-amniotic nutrient administration at E17, and 6 embryos were randomly selected from each treatment group at E19 (48 h post intra-amniotic nutrient administration). Post-hatch tissue sampling was conducted on 6 randomly selected chicks from each treatment group at hatch, day 3 post-hatch (D3) and D7. Embryos were euthanized by cervical dislocation and chicks were euthanized by CO_2_. The jejunum segment of their small intestines (a ≈1 cm piece from the midpoint between the duodenal loop and Meckel’s diverticulum) was excised, lumen contents were flushed out with 4 °C phosphate buffered saline (PBS) and tissues were divided into three segments. The first segment was flash frozen in liquid nitrogen and stored at -80 °C for RNA extraction; the second segment was fixed in 3.7% formaldehyde in PBS at pH 7.4 (Sigma-Aldrich, Rehovot, Israel) for 24 h at RT for immunohistochemistry; the third segment was fixed in 2% glutaraldehyde and 4% formaldehyde in 0.2 M CaCo buffer at pH 7.4 (Sigma-Aldrich) for 24 h at RT for scanning electron microscopy.

### Immunohistochemistry

Following fixation, tissues were dehydrated in graded series of ethanol, cleared by Histochoice® (Sigma-Aldrich, Rehovot, Israel) and embedded in Paraplast® (Sigma-Aldrich). Tissue blocks were sectioned 5 µm thick and mounted on SuperFrost Plus™ glass slides (Bar-Naor Ltd., Petah-Tikva, Israel). Tissues were deparaffinized by Histochoice® (Sigma-Aldrich) and rehydrated in a graded series of ethanol. Antigen retrieval was performed in boiling citrate buffer at pH 5.5 (Sigma-Aldrich) for 20 min. Tissues were permeabilizied with 0.1% Tween® (Sigma-Aldrich) in PBS (PBST). Following incubation in a blocking solution of 1% bovine serum albumin (BSA) (Sigma-Aldrich) in PBS, tissues were incubated overnight at 4 °C with primary antibodies: for E-cad-Villin immunostaining: mouse anti-E-cad (1:250, 610182, BD Bioscience, NJ, USA) and rabbit anti-Villin (1:100, ab130751, Abcam, MA, USA); for GLP-2 immunostaining: rabbit anti-GLP-2 (Arg34) (1:500, H-028-14, Phoenix Pharmaceuticals, CA, USA). Tissues were then washed in PBS and PBST, and incubated for 1 h at RT with the following secondary antibodies: donkey anti-mouse Cy3 (1:100, 715-165-150, Jackson ImmunoResearch Laboratories, PA, USA) for E-cad detection, and donkey anti-Rabbit Alexa Fluor 488 (1:100, 711-545-152, Jackson ImmunoResearch Laboratories, PA, USA) for Villin and GLP-2 detection. Tissues were then washed in PBS and incubated with 4′,6-diamidino-2-phenylindole (DAPI) (1:1000, Sigma-Aldrich) for 5 min, followed by washing in distilled water (DW). Slides were mounted using Fluorescent mounting medium (GBI Labs, WA, USA). Images were acquired using a Zeiss Axio Imager M1 microscope and an AxioCam MR3 camera with ZEN 2.3 (blue edition) software (Zeiss, Gottingen, Germany). Measurements of cell lengths and fluorescence intensity were conducted using FIJI software.

### Scanning electron microscopy

Following fixation, tissues were washed three times with 0.2 M CaCo buffer, followed by post-fixation in 0.1% OsO4 (Sigma-Aldrich, Rehovot, Israel) in 0.2 M CaCo buffer for 1 h. Tissues were then washed three times with 0.2 M CaCo buffer and four times in DW, dehydrated in 20%, 30%, 50%, 70%, 90% 100% ethanol, and dried in a K850 Critical Point Dryer (Quorom Technologies Ltd., East Sussex, UK). Tissues were then mounted on aluminum stubs with carbon tape, villi tips were trimmed off using a razor blade under a stereomicroscope for visualization of microvilli lengths, and all stubs were sputter-coated with iridium (Q150T ES Quorom Technologies Pvt. Ltd., East Sussex, UK). Visualization was conducted using a JEOL 7800F high-resolution scanning electron microscope (Jeol Ltd., Tokyo, Japan), at 3kv and 4 WD. Measurements and figure preparation were conducted using FIJI software.

### Real-time quantitative polymerase chain reaction

Total RNA was extracted from frozen tissues using Trizol reagent (Sigma-Aldrich, Rehovot, Israel) according to the manufacturer’s protocol. cDNA was synthesized from 1.0 μg total RNA using a PCRBIO 1-Step Go RT-PCR Kit (Tamar, Mevaseret Zion, Israel). Primer sequences were taken from previous studies for PepT-1 (accession no. NM_204365.1)^34^, IGF-1 (accession no. NM_001004384.2)^52^ and β-actin (accession no. NM_205518.1)^47^. For all other target genes, custom primers were designed using Primer Blast (https://www.ncbi.nlm.nih.gov/tools/primer-blast/) and listed in Table 1. All primers span exon-exon junctions, and were validated for exclusion of genomic DNA contamination on pooled chicken cDNA and genomic DNA samples by 1.5% agarose gel electrophoresis.

**Table 1:** Primer list for real-time qPCR.

Gene	Accession number	Forward primer (5′)	Reverse primer (3′)	Amplicon length
SGLT-1	NM_001293240.1	AGCGAGTAAATGAGCAGGGTG	CCCACAGATGATGAACGGACA	137
TJP-1	XM_015278980.2	AGGTGAAGTGTTTCGGGTTG	CTTCCAAAGACAGCAGGAGG	171
TJP-2	NM_204918.1	TGAACCACCAAAGGGGAAGTT	ATTTCTCAGGATGGGACGCC	175
Occludin	NM_205128.1	AGCGCTACAAGCAGGAGTTC	TCCTCTGCCACATCCTGGTAT	151
PCB	NM_205260.5	GTTCAAGGCAGCTGGCAAAA	ATGTGCCTTGTGAATGACGC	130
GLP-2R	NM_001163248.1	ATACTTTTTGGTCTCTGCTCCAG	CAGAGATCCTTTGGCCTGTT	108
IGF-1R	NM_205032.2	ACCTTGTTGTGCTTGTCCCA	CTTCCGATCAGGTCTGGGGA	124

Real-Time quantitative polymerase chain reaction (qPCR) was conducted in a Lightcycler® 96 instrument (Roche Diagnostic International, Zurich, Switzerland). Reactions were composed of 3.0 μL cDNA, diluted 1:25 in ultra-pure water (UPW), 1 μL of each primer (4 μM), 5 µL UPW and 10 μL Fast SYBR™ Green Master Mix (Sigma-Aldrich). A standard curve was generated for target and reference genes, assuring R^2^ values of > 0.9 and primer efficiencies of 2 ± 0.1. All reactions were performed in duplicates and a non-template control was run for each primer pair. Reactions consisted of pre-incubation at 95 °C for 1 min, 40 cycles of 2-step amplification (95 °C for 10 s and 60 °C for 30 s), and a melting curve analysis (95 °C for 10 s, 65 °C for 60 s and 97 °C for 1 s) for confirming single product amplification. Expression levels were calculated using the 2^−ΔΔCt^ method^53^. Expression of all examined genes was normalized to β-actin, and changes in expression between groups at each timepoint were relative to mean ΔCt values at E17 for SGLT-1, PepT-1, TJP-1, TJP-2 and Occludin, and relative to mean ΔCt values in the Control group at each timepoint for PGB, GLP-2R, IGF-1, and IGF-1R.

### Statistical analysis

Data was analyzed using JMP Pro version 15.0 (SAS Institute, NC, US). In each analysis, equal variances were validated by Levene and Bartlett tests. Data was analyzed by one-way ANOVA at each timepoint and mean comparisons between each treatment group and the Control group were performed by t-test with significance set at *P* < 0.05. Graphical data are presented as means ± standard error means and significant differences are represented by asterisks: *P < 0.05, **P < 0.01, ***P < 0.001 ****P < 0.0001.
